# Overexposed and Understudied: Environmental Risks Among Older Adults Experiencing Homelessness in Phoenix, Arizona

**DOI:** 10.1029/2025GH001372

**Published:** 2025-05-12

**Authors:** Zachary Van Tol, Ariane Middel, Jennifer K. Vanos, Kristin M. Ferguson

**Affiliations:** ^1^ School of Sustainability Arizona State University Tempe AZ USA; ^2^ School of Arts Media and Engineering Arizona State University Tempe AZ USA; ^3^ School of Social Work Arizona State University Phoenix AZ USA

**Keywords:** heat, air pollution, homelessness, environmental risks

## Abstract

Individuals experiencing homelessness are highly vulnerable to urban environmental hazards, such as heat and air pollution, due to a lack of stable housing and limited access to indoor or cooled spaces. These risks are heightened for older adults and individuals with preexisting health conditions. With intensifying summer heat in the American Southwest and the persistence of urban homelessness, this study explores how older adults experiencing homelessness in Phoenix, Arizona perceive and interact with heat and air pollution as well as the adequacy of available coping resources and information. A survey was co‐produced with community members with lived experience. A sub‐group of community members assisted in proctoring the surveys among adults 55 and older at a downtown service agency. Survey results informed the location of data collection using MaRTy—a mobile biometeorological cart—and remotely sensed fine particulate matter (PM_2.5_). Findings reveal that heat and air pollution significantly influence travel decisions and contribute to health issues for many respondents. Midday and evening thermal radiation levels regularly exceeded safe thresholds, while PM_2.5_ concentrations often surpassed annual air quality guidelines, though they posed less acute health risks. Most participants reported awareness of health risks and employed strategies to reduce exposure. However, many expressed that city‐level, public resources are insufficient to prevent heat‐ and air pollution‐related health issues. Findings underscore the need for targeted interventions—such as better access to transportation and essential healthcare and extended hours of operation for service providers—to enhance resource accessibility and mitigate environmental health risks for vulnerable populations.

## Key Points

- Heat and air pollution significantly impact travel and health for older adults experiencing homelessness in Phoenix, Arizona
- Measured thermal exposures exceeded safe thresholds; particulate matter may pose long‐term risks for those with chronic health conditions
- Expanded transit, health care, and provider operating hours were identified as potential ways to reduce environmental health risks

## 1 Introduction

Homelessness is an omnipresent issue in many American cities. A confluence of complex factors, such as housing unaffordability, the drug epidemic, and mental health crises, have led to more than 650,000 Americans experiencing homelessness on any given night in 2023, with the majority doing so in large cities (de Sousa et al., 2024). Largely due to its perennial sunshine and relatively mild winters, cities across the United States Southwest are home to a disproportionate population of people experiencing homelessness (de Sousa et al., 2024). One such city is Phoenix, Arizona—home to the state's capital and over 1.6 million people. Phoenix is the largest city in Arizona and the fifth largest in the United States (U.S. Census Bureau, 2024). An estimated 2,701 Phoenicians were experiencing unsheltered homelessness at the time of the 2024 Point‐in‐Time (PIT) count, with nearly 9,500 people experiencing homelessness in the Maricopa Regional Continuum of Care, a broader geographic area that encompasses a network of service providers working to end homelessness (MAG, 2024).

A warmer winter climate may attract many, but it comes with its share of hazardous tradeoffs in the summer. Air temperatures in Phoenix consistently exceed 100°F (37.8°C), sometimes not dropping below 90°F (32.2°C) at night during the long summer months. Simultaneously, the area's extreme heat and intensive anthropogenic activities contribute to poor air quality by intensifying the formation of ground‐level ozone and concentration of particulate matter (PM) (Grineski et al., 2007). The combination of these hazards can prove consequential for the health of many who are unable to avoid prolonged exposure (Van Tol et al., 2024). Among others, heat can increase cardiovascular strain by reducing the heart's filling pressure while simultaneously requiring the heart to pump faster (i.e., higher heart rate, higher cardiovascular strain) (Ebi et al., 2021). Similarly, pollutants interact with the cardiovascular system, potentially raising blood pressure, altering the autonomic nervous system, causing inflammation, and restricting blood flow to the heart (Vallero, 2014). These concerns are amplified for older adults, individuals with substance use disorders, and those with preexisting health conditions (Geller & Zenick, 2005; Kenney et al., 2014; Vickery, 2018). For example, Analitis et al. (2014) found that deaths during heat waves were 54% higher on days with elevated ozone and 36% higher on days with elevated PM for those ages 75–84 across nine European cities between 1990 and 2004. Further, medications, illicit drugs, and alcohol can exacerbate adverse physiological responses to environmental stressors by causing dehydration, electrolytic disorders, and/or interference with the body's thermoregulation processes (e.g., Cheshire & Fealey, 2008; Crank et al., 2023; Marco et al., 2021; Martin‐Latry et al., 2007). In Maricopa County, where Phoenix is situated, roughly 21% of people experiencing homelessness are age 55 or older, and approximately 28% self‐report a mental illness or substance abuse issue (MAG, 2024).

When considering these environmental hazards—extreme heat and air pollution—it is important to recognize the differences in exposure pathways and temporal implications of exposure, particularly for those experiencing homelessness. Heat exposure primarily leads to acute health outcomes, including heat stroke, exhaustion, or dehydration (Petkova et al., 2014), while prolonged exposure can contribute to chronic conditions like renal failure (Fletcher et al., 2012). In contrast, air pollution—especially at the levels seen in Phoenix—is predominantly associated with exacerbating chronic respiratory diseases such as chronic obstructive pulmonary disease, though acute issues, like asthma attacks or respiratory failure for those with pre‐existing respiratory conditions, may also occur depending on exposure type and/or particle size (Litchfield et al., 2010; Schwarze et al., 2006). Within the given population, longer periods outdoors exacerbate what may be experienced by the general population for both hazards due to alternative time activity patterns that impact the exposure pathway (e.g., Kuras et al., 2017). Yet the differences in health impact between the two exposures are important to point out, as heat has a much more immediate onset of adverse effects—especially for vulnerable groups—often leading to heat strain and hospitalization within the same day (Ebi et al., 2021), which has been demonstrated in the population experiencing homelessness as well (Lin et al., 2024; Schwarz et al., 2022). In comparison, the more serious health impacts of air pollution are less well‐defined and believed to develop over much longer periods of time (Henning, 2024).

Access to climate‐friendly spaces is often cited (e.g., Eisenman et al., 2016) as paramount to the fight against environmentally exacerbated morbidity and mortality. Access to such spaces is mediated through a variety of avenues, including stigmatization, physical infrastructure, policing, and policies, which can leave those most at risk without adequate protection (Amster, 2008; Cress & Snow, 2000; Mitchell, 2003). In attempts to increase access to climate‐safe spaces, Phoenix heavily relies on a network of community partners. The established the Heat Relief Network (HRN) in 2005, an effort that created spaces to cool off across the City of Phoenix (Harlan et al., 2019). HRN sites are indoor spaces operated by community‐based organizations that offer water, cooling, and donation sites intended to prevent heat‐related illnesses and deaths (Heat Relief Network, 2024). These centers are open to the broader public, but their availability and functionality are perhaps most consequential for people experiencing homelessness within the community (Hondula et al., 2024). Although the HRN was created in response to heat, access to air‐conditioned spaces simultaneously creates the opportunity for air filtration interventions for air pollution (e.g., Huang et al., 2023). During the summer of 2023, such facilities provided relief to tens of thousands of people (Maricopa County Department of Public Health & Infomatics, 2024).

More recently, the City of Phoenix created an alternative to traditional homeless encampments in what they call the “Safe Outdoor Space” (SOS; City of Phoenix, 2023). The Safe Outdoor Space (SOS) leveraged an available indoor and outdoor space with access to utilities, enabling extensive wrap‐around services. Although not technically classified as a shelter or referred to as a campsite, the SOS is generally a large, outdoor campground that provides individuals experiencing unsheltered homelessness with a safe space to sleep semi‐sheltered from the elements (thanks to a large awning covering tents affixed to a turf lawn and a small indoor eating space with evaporative cooling) without having to separate from their social networks, pets, or belongings. Despite such intervention, 2023 saw record heat deaths, with 645 heat‐related deaths documented across Maricopa County (340 of such deaths occurred in Phoenix city limits), of which people experiencing homelessness accounted for 45% (Maricopa County, 2024). Further, the preliminary numbers for 2024 indicate that heat‐related deaths could once again surpass 600 upon completion of cases under investigation (Maricopa County, 2024). With dire consequences, the distribution, availability, and use of such resources are paramount during the summer months, especially for unhoused community members.

While urban homelessness and environmental hazards are often studied independently, limited attention has been given to how older adults experiencing homelessness perceive and cope with these dual threats in the context of an intensifying urban climate crisis (see Noor et al., 2025). Some studies have examined environmental conditions in the spaces people experiencing homelessness spend the bulk of their time (e.g., Karanja et al., 2023), while others have quantified potential exposures along frequented routes to account for the heightened mobility typical of this population (e.g., Longo et al., 2017). This mixed‐method study seeks to understand how older adults experiencing homelessness (ages 55+ years) perceive and interact with their surroundings, particularly heat and air pollution. We couple qualitative and quantitative data to determine whether the current support mechanisms effectively address the specific needs of older adults experiencing homelessness, as it is suggested to be the most representative approach for gathering insights on human‐environmental reactions (e.g., Longo et al., 2017; MacMurdo et al., 2022; Sanchez, 2011). The following research questions guide this study: (a) What are the thermal and air pollution exposures along the routes frequented by older adults experiencing homelessness in Phoenix, Arizona; (b) how do this group's heat and air pollution perceptions align with the measured risks; and, (c) do city resources and awareness campaigns about heat and air pollution, aimed at people experiencing homelessness, adequately meet their needs? Findings are most immediately impactful for service providers through organizational changes, while also holding insights for policymakers and urban planners to lessen the environmental impacts on a vulnerable portion of the populace through policy and other structural changes.

## 2 Methods

### 2.1 Surveys

A survey was developed to assess perceptions of environmental hazards—specifically heat and air pollution—and attitudes toward coping resources and behaviors among older adults experiencing homelessness in Phoenix, Arizona. The study involved two establishments: Community 43, which assisted in designing the study, and Justa Center, where participants answered the survey. Community 43 is a clubhouse‐style outpatient clinic in central Phoenix that serves individuals with severe mental illness, while Justa Center, a local non‐profit, provides life‐sustaining resources and support for seniors (55+ years old) experiencing homelessness. A cohort of members of Community 43, who self‐identified as having past experience with housing difficulties, contributed their expertise during two working sessions in March 2024, helping to create and refine survey questions. Although they could not take the survey—as they were not actively experiencing homelessness—their lived experiences greatly enriched both the study design and its findings. The survey comprised 51 questions across the following categories: demographic and behavioral, movement/transportation, climate perceptions, resource availability, and local knowledge mapping. When appropriate, questions were designed using a Likert scale (Bernstein, 2005) to allow for a nuanced evaluation of respondents' attitudes (for the survey in its entirety, see Supporting Information S1). The finalized surveys were digitized using Qualtrics to enable data collection and recording via tablets. Justa Center helped to recruit survey participants (i.e., community members actively experiencing homelessness) and further embedded our research within the community.

A total of 40 individual surveys were conducted over two days in June 2024. On these days, outdoor temperatures reached 42°C (107.6°F) and 45°C (113°F), respectively. Surveys were conducted indoors in a cooled, private room. Survey participants were limited by the following criteria: (a) aged 55 years or older; (b) English‐speaking or understanding; and (c) self‐identified as homeless. Four members of Community 43 administered the surveys in teams of two, providing support such as answering questions and assisting with reading. They also provided compassionate listening to participants and distributed informative flyers regarding the region's HRN and heat safety. Participants were compensated with a $25 grocery gift card for their time, and members of Community 43 were fairly compensated for their consulting and surveying time, with the amount decided in consultation with organizational leadership. The findings outlined throughout this study were shared with leadership and community members at Community 43 and Justa Center. All procedures, including informed consent, were approved by the Institutional Review Board of Arizona State University (IRB approval number: STUDY00018399).

### 2.2 Biometeorological Data Collection

Leveraging the survey results to establish areas of interest, this study employed MaRTy. This mobile biometeorological cart measures mean radiant temperature (MRT), air temperature (*T*_air_), relative humidity (RH), wind speed, and wind direction at pedestrian height at 2‐s intervals (Middel & Krayenhoff, 2019). Taking into consideration short‐ and long‐wave radiation, MRT represents the summation of radiant heat exchange between an individual and their environment, including shade, the underlying surface, and other surrounding materials, allowing for hyper‐local, human‐relevant thermal comfort metrics (Johansson et al., 2014; Oke, 2017). Meteorological variables were collected and paired with GPS coordinates along a route (and at stops) as determined by the local knowledge mapping section of the survey. Approximately 20 s of data were discarded from the start and end of each stop to account for the response time of MaRTy's net radiometers—values were averaged and time detrended to limit error. Data were collected in the morning, mid‐day, and evening to provide a diurnal profile of exposures. Meteorological data collection was determined based on weather conditions, aiming to measure a day most representative of Phoenix summers—hot, dry, and sunny. Our collection day (20 August 2024) was simultaneously representative of our survey days, as air temperatures maxed out at just under 110°F (43.3°C) and winds remained calm.

### 2.3 Particulate Matter Modeling

In addition to thermal metrics, we utilized estimated particulate matter (PM_2.5_) concentrations to quantify the air pollution risk to survey participants. As a pollutant class that contains an amalgam of other pollutants (e.g., elements of combustion byproducts, heavy metals, and organic compounds; see Zhang et al., 2015), PM_2.5_ serves as a useful indicator of general air pollution concerns for this exploratory, first‐of‐its‐kind study. Since ground monitoring sites are often sparse and unevenly distributed, relying solely on ground‐based PM_2.5_ data may not accurately represent urban air quality. Urban areas also exhibit highly differential air pollution gradients, depending on city design and proximity to emitters (Pinto et al., 2004). Therefore, we use satellite‐based data to model PM_2.5_ concentrations (μg/m^3^) at an ultrahigh, 100‐m spatial resolution.

To build the model, meteorological data, including temperature, surface pressure, wind speed, and wind direction, were acquired from ERA5‐Land (Muñoz Sabater, 2019). These factors influence the movement and chemical reactions of PM_2.5_ particles. For example, temperature drives secondary aerosol formation, while wind patterns determine dispersal routes. To downscale the ERA5‐Land data (from 9 km to 100 m), we employed bilinear interpolation, following established methodologies in previous studies (Shetty et al., 2025; Wei et al., 2023; Wu et al., 2024; Yang et al., 2020). Phoenix is a relatively flat urban area with minimal terrain complexity and no extensive forest coverage, reducing the likelihood of localized variations in air temperature due to topographical effects. Thus, bilinear interpolation provides a reasonable approximation of the meteorological background at finer scales. To further enhance the fine‐scale characterization of PM_2.5_ distribution, we incorporated high‐resolution land cover data (Normalized Difference Vegetation Index (NDVI)) and nighttime light data (NTL) data, which capture local variations in land use and human activities (see Feng et al., 2022; Li et al., 2017). These data sets (see Table S1 in Supporting Information S1), in conjunction with in‐situ PM_2.5_ measurements, help constrain and refine our final estimates.

Hourly PM_2.5_ concentrations were obtained from 9 U.S. EPA federal reference method samplers in Phoenix for the week of August 19th–25th. These ground measurements provide accurate, localized pollution data and serve as the foundation for training and validating our model. These data were downloaded from the EPA's Air Quality System Technology Transfer Network. A support vector regression model with a radial basis function kernel was used to link our inputs to the ground‐level EPA site PM_2.5_ measurements. The model demonstrated strong performance, achieving an *R* ^2^ value of 0.82, indicating a high level of agreement between predicted and observed PM_2.5_ concentrations.

## 3 Results

### 3.1 Survey Responses

We surveyed 40 people aged 55 and older, with a mean age of 62 and birth years ranging from 1947 to 1969. The majority of respondents (32) identified themselves as cisgender male, while eight identified as cisgender female, and no one self‐identified as transgender or non‐binary. The group was racially/ethnically diverse, with 20 people identifying as White, 12 as Black or African American, three as Hispanic or Latino, three as mixed race, and two as Native American or Indigenous. One‐quarter of respondents were native Phoenix residents (i.e., born in Phoenix), whereas 30 were born in a different city. Half of the survey pool acknowledged struggling with housing for at least a year, with 15 people facing housing complications for months, four for weeks, and only one individual struggling with housing for less than a day.

#### 3.1.1 Health

Physical ailments were far more common among respondents than mental health challenges. Yet, participants reported equal difficulty in receiving medication for both mental and physical ailments (Table 1). The majority of the survey pool—36 of the 40 surveyees (90%)—reported having health insurance, with Medicaid through the Arizona Health Care Cost Containment System the most popular coverage among respondents (17, 42.5%). Other popular responses include Mercy Care (8, 20.0%) and United Health Care (3, 7.5%). Despite a large proportion of participants having coverage, 8 (20.0%) individuals reported insurance as a barrier to accessing services for both mental and physical health conditions, with two additional people reporting health insurance as a barrier to services for either physical or mental health conditions but not both (leaving 75% unencumbered by insurance when accessing necessary services). Just as concerning, 22.5% of respondents reported having a physical health condition or diverse ability that impacts their access to services for either mental health conditions (2), physical health conditions (3), or both (4).

**Table 1:** Survey Respondents' Relationship With Physical and Mental Health in Percentage and (Count)

Question	Yes	No	No Condition/Illness
Have you had a psychiatric analysis or diagnosis of any mental illnesses?	37.5% (15)	62.5% (25)	—
Are you able to access necessary medication if you are dealing with a mental illness?	62.5% (25)	7.5% (3)	30.0% (12)
Have you been diagnosed with any physical health conditions?	65.0% (26)	35.0% (14)	—
Are you able to access necessary medication if you are dealing with a physical health condition?	80.0% (32)	7.5% (3)	12.5% (5)

Although the literature reports that substance use among those experiencing homelessness often exceeds that of the housed population (Morrison Institute for Public Policy, 2022; National Coalition for the Homeless, 2017; Polcin, 2016), our results do not reflect this (Table 2). Just four individuals (10.0% of respondents) reported using alcohol or other substances to cope with experiencing homelessness. Perhaps more telling, when asked whether or not an addiction limits their access to services, three people indicated “yes,” 33 selected “no,” and only four opted for the selection “I do not have an addiction.” These findings match what has been reported by local government agencies (see Maricopa County Department of Public Health & Infomatics, 2024).

**Table 2:** Self‐Reported Alcohol and Drug Use Among Respondents in Percentage and (Count)

Consumption	Never	Rarely	Sometimes	Almost every day	Frequently
Alcohol	55.0% (22)	30.0% (12)	10.0% (4)	5.0% (2)	—
Illegal or illicit drugs	85.0% (34)	10.0% (4)	5.0% (2)	—	—

#### 3.1.2 Mobility

Our survey population displayed diverse modes of transportation, but walking was by far the most common method for getting around, as it was selected 21 times (52.5%) by survey respondents. Taking the bus is the next most common method (15 times) for navigating Phoenix, followed by biking (5), a car (4), and a train (1) (some respondents provided a list of two or three methods of transportation in the “other” section; we opted to retain each unique response). The survey revealed that adequate transportation greatly impacts respondents' ability to seek necessary services, with only 11 individuals selecting that transportation is always available when they want to seek out necessary services (Table S2 in Supporting Information S1). Further complicating matters is the need to account for one's belongings, as 62.5% (25) of respondents indicate that they carry their items around with them, and only 37.5% (15) signify that they have a safe place to store their belongings. Despite this limitation, few surveyees indicated that their belongings are perennial obstacles when seeking the services they need (Table S2 in Supporting Information S1). Out of our population, 45.0% (18) claim their belongings are never a limitation to service access, and only 10.0% (4) selected that their belongings always limit their ability to access necessary services.

Participants were asked about the primary reason they moved from place to place, with five options—climate, food or water, health services, policing, safety—and an option to select “other” and provide a reason. Out of the five predefined options, policing was the most common response, chosen by eight participants (20.0%), followed by climate (7, 17.5%), food or water and safety (5, 12.5%), and health services (1, 2.5%). Fourteen respondents chose “other”, with unique reasonings provided. These responses were as follows: all of the above (4), expenses (2), boredom (2), all except policing (1), safety and policing (1), staying away from danger (1), looking for affordable housing (1), does not apply (1), and blank (1). When asked how many hours they spend outside, unshaded on any given day, the average response was roughly 7 hours, with a quarter of the respondents spending 10 or more hours a day outside and unprotected from the sun. However, given the range of answers (i.e., five respondents indicating hours >12), the responses are better interpreted as older adults experiencing homelessness in Phoenix spend considerable time outside in any capacity, irrespective of shading (i.e., little can be drawn in terms of when/where individuals spend time outside).

Owing to the elevated amount of time spent outdoors, it is important to understand this population's relationship with local weather when determining where they move during the day. Common among the respondents' answers is that heat is more of a determinant of movement than air pollution during the day and night (Table 3). Roughly the same number of individuals (approximately one‐third in each category) indicated that air pollution always or never determines where they go during the day and night, indicating a stark divide in the impact of air quality on daily life. In contrast, there is a six‐fold difference in the number of individuals who indicated that their movement is “never” (4) versus “always” (24) influenced by heat during the day, that difference dissipates at night. Notably, we see a 12.5% increase in respondents whose movement is never impacted by the heat at night relative to their responses for the daytime hours.

**Table 3:** Role of Heat and Air Pollution in the Movement of Older Adults Experiencing Homelessness in Percentage and (Count)

Survey Question	Never	Rarely	Sometimes	Often	Always
Does heat determine where you go during the day?	10.0% (4)	—	7.5% (3)	22.5% (9)	60.0% (24)
Does air pollution determine where you go during the day?	32.5% (13)	12.5% (5)	15.0% (6)	12.5% (5)	27.5% (11)
Does heat determine where you decide to sleep?	22.5% (9)	—	15.0% (6)	12.5% (5)	50.0% (20)
Does air pollution determine where you decide to sleep?	35.0% (14)	—	20.0% (8)	10.0% (4)	35.0% (14)

#### 3.1.3 Heat and Air Pollution Awareness

**Figure 1:**
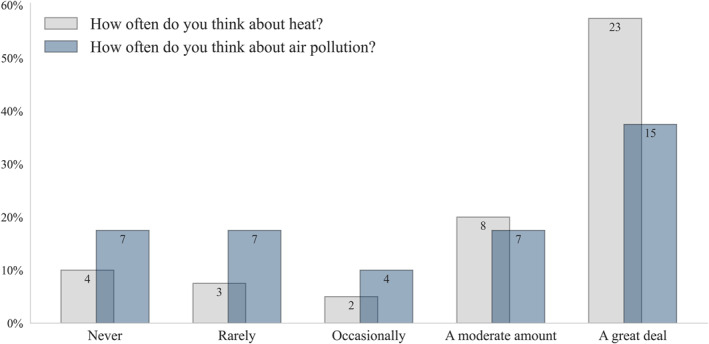
Frequency of thinking about heat and air pollution among survey respondents. The bar plot shows the percentage of responses for each category, ranging from “never” to “a great deal.” The bars' labels indicate the number of responses within each category. For example, 57.5% of respondents think about the heat a great deal, corresponding to 23 responses, while 37.5% think about air pollution a great deal, corresponding to 15 responses.

Older adults experiencing homelessness think about heat and air pollution often (Figure 1). Nearly 78% of respondents indicated that they think about heat more than occasionally, compared to 55% who think about air pollution more than occasionally. Over one‐third of respondents rarely or never think about air pollution, while just over one‐sixth never or rarely have thoughts about heat. The discrepancy between thinking about heat and air pollution could reflect time spent outdoors; perhaps those outside for more prolonged periods are more aware of the impacts. Of the 14 respondents who selected that they think about both heat and air pollution “a great deal,” the average amount of time spent outside was 9.4 hr per day; this time is compared to roughly 7 hr for the four individuals who claim that they “never” think about heat or air pollution.

#### 3.1.4 Health and Environment

To further break down the relationship between older adults experiencing homelessness and heat and air pollution, we asked surveyees about their knowledge of health risks related to these climate variables (Table 4). Responses indicate that most of the survey population is at least “somewhat” aware of the health risks related to air pollution and heat. Of those surveyed, 65.0% (26) indicated that they believe the environment has a moderate or extreme impact on their health. Additionally, a quarter of individuals surveyed expressed no to slight knowledge about the impacts of air pollution on their health, and a fifth of respondents indicated that they believe the environment only has a slight or no impact on their health.

**Table 4:** Awareness of Health Impacts From Heat and Air Pollution in Older Adults Experiencing Homelessness in Percentage and (Count)

Survey Question	Not at all	Slightly	Somewhat	Moderately	Extremely
Do you feel like your environment has an impact on your health?	12.5% (5)	7.5% (3)	15.0% (6)	17.5% (7)	47.5% (19)
How aware are you of human health risks associated with heat?	5.0% (2)	—	15.0% (6)	22.5% (9)	57.5% (23)
How aware are you of human health risks associated with air pollution?	15.0% (6)	10.0% (4)	5.0% (2)	17.5% (7)	52.5% (21)

**Figure 2:**
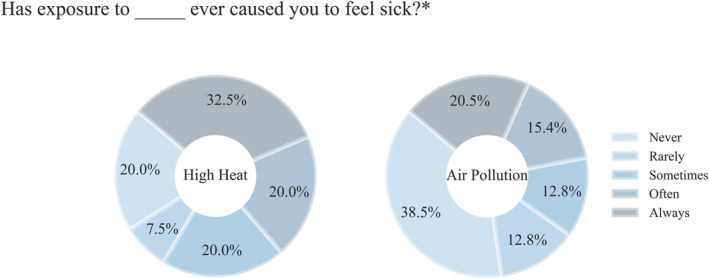
The proportion of survey respondents who recall heat or air pollution causing them to feel sick. *39 of 40 survey participants responded to these research questions.

A higher proportion of respondents recall feeling sick from the heat compared to air pollution, with more than half remembering heat “often” or “always” causing them to feel sick compared to just over a third recalling the same feelings caused by air pollution (Figure 2). In line with awareness of the health risks related to heat and air pollution (Table 4), more than a quarter of surveyees believe that high heat exposure has rarely or never caused them to feel sick and more than 50.0% feel that way about air pollution. Regardless, these numbers are concerning, as only 25.0% (10) of surveyees indicated they can always avoid the heat when they wish to, and 17.5% (7) selected that they cannot avoid the heat when they want to. Interestingly, when asked the same question about air pollution, 32.5% (13) indicated that they can never avoid it when they want to and only 17.5% (7) selected that they can always avoid air pollution when they want to.

#### 3.1.5 Survival Tactics

The open‐ended question of “How do you survive the heat and air pollution?” in the Arizona summers revealed a rich picture of the diverse coping strategies for staying safe (see Figure S1 in Supporting Information S1). The top of the list was finding shade, which was mentioned 11 times in responses, followed by water (5), staying inside (4), and support systems like Justa Center (4) and Central Arizona Shelter Services (4). Overall, survey respondents were split when asked whether or not the community provided enough resources to help them avoid exposure to heat and poor air quality (Table S3 in Supporting Information S1). Opinions of resource provisions fared better when asked about the heat relative to the same question about poor air quality. Of the 40 participants, less than a fifth of respondents strongly agree that the community provides enough resources to avoid the heat, with 7.5% feeling that way about poor air quality. More problematic is the disproportionate number of individuals at the other end of the spectrum who strongly disagree with the sentiment that the community is providing enough resources. In addition to these perspectives, just under half of respondents (47.5%) said that seasonal weather conditions impact their access to services, while 35% said there is no seasonal variation, and seven individuals indicated they are unsure.

**Figure 3:**
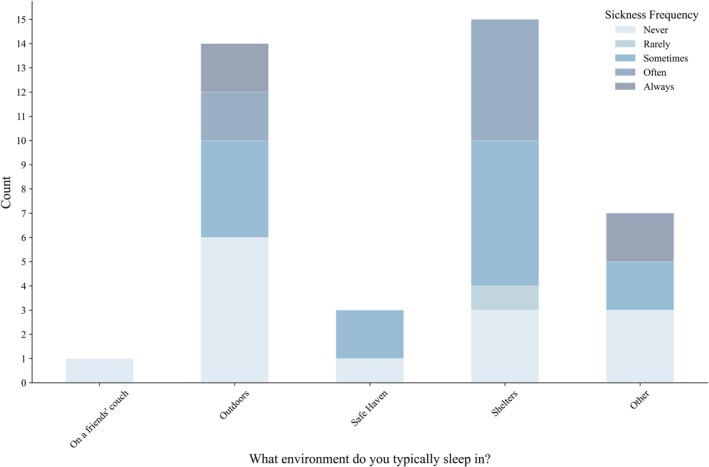
This stacked bar chart displays the relationship between respondents' sleeping environment and feelings of sickness. Each bar represents the number of respondents who typically sleep in that specific environment, while each color indicates the associated frequency of sickness while sleeping (e.g., two respondents typically sleep outdoors where they “always” feel sick).

Temperatures overnight are often cited as a leading determinant of the body's ability to recover from the exposures of the day (Seltenrich, 2023). As such, we asked surveyees where they typically slept—to garner an approximation of overall environmental exposure—followed by a self‐reported, subjective response on how often their environment contributed to feelings of sickness. A substantial 35% (14) of respondents indicated that their typical sleeping arrangements never make them feel sick (Figure 3). Sleeping locations reported as the most likely to make survey respondents feel sick at least “sometimes” were outdoors and in shelters. Seven respondents selected “other,” and responded with various answers, including Another area worth highlighting is shelters, which led the way in terms of responses and also has a more common relationship with feelings of sickness. This relationship could result from climate and proximity to other individuals, which has been seen to expedite the transmission of communicable diseases (e.g., Coffey et al., 2009; Martin et al., 2014; McCulloch et al., 2023).

#### 3.1.6 Safe Outdoor Space

It is worth prefacing our results with the fact that 52.5% of respondents, or 21 surveyees, indicated that they are “not at all” aware of the SOS opened by the City of Phoenix in the summer 2024, and only 14 people surveyed indicated that they are “moderately” or “extremely” aware of the SOS. Further, only five people, or 12.5% of the respondents, indicated that they used the SOS at the time of the survey (June 2024). Despite these findings, after a brief explanation of the SOS from surveyors, the majority of survey respondents (21) indicated that they wanted to use the SOS, with 14 selecting that they did not want to use the SOS, three people preferring not to say, and two others showing potential interest in the “other” section. We proceeded to garner perspectives from the participants with four additional statements regarding the SOS (Table 5). Although base knowledge/awareness surrounding the SOS was low amongst this group, very few respondents disagreed or strongly disagreed with the positive statements we raised.

**Table 5:** Perceptions of the Safe Outdoor Space Amongst Older Adults Experiencing Homelessness in Percentage and (Count)

Survey Statements	Strongly disagree	Disagree	Neither agree nor disagree	Agree	Strongly agree
The SOS is a positive change.	2.5% (1)	7.5% (3)	15.0% (6)	62.5% (25)	12.5% (5)
The SOS provides enough freedom to those who stay there.	2.5% (1)	17.5% (7)	32.5% (13)	37.5% (15)	10.0% (4)
The SOS provides all the resources necessary to stay healthy.	5.0% (2)	17.5% (7)	35.0% (14)	37.5% (15)	5.0% (2)
The SOS provides adequate shade and access to air conditioning.	2.5% (1)	12.5% (5)	45.0% (18)	35.0% (14)	5.0% (2)

The most interesting takeaway from these statements is that three‐quarters of respondents either agree or strongly agree that the SOS is a positive change. Given the low awareness regarding the SOS, we see a shift in responses toward more neutral feelings as the questions get more specific. What is more telling from our survey results is the responses when asked what the SOS could provide to improve their quality of life. This open‐ended question led to 23 responses, ranging from housing and healthcare to shade and cooling (A/C). In addition to these popular responses, surveyees listed better caseload management, more services for housing, healthcare services, and more shelters.

### 3.2 Potential Environmental Exposures

**Figure 4:**
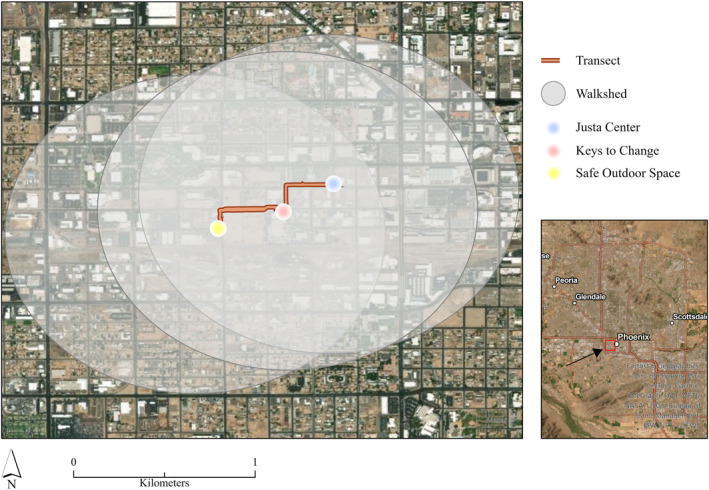
Walking transect on Tuesday, 20 August 2024 using the biometeorological cart, MaRTy, based on responses to our survey question (see Figure S3 in Supporting Information S1). A reasonable walkshed (0.875‐km in any direction) for survey participants is signified for each location by the light gray buffer.

With a better understanding of awareness surrounding heat and air pollution impacts for older adults experiencing homelessness, we wanted to collect informed measurements of the potential health impacts. As such, our survey included questions designed to identify places for in‐situ meteorological measurements. We asked surveyees to select the image that is most similar to where they spend most of their time (see Figure S2 in Supporting Information S1). Shelter space was overwhelmingly the most popular, with 19 selections. Next were parks (10), followed by parking lots (3), abandoned houses (3), wetlands (2), and the canals (1). Two others chose to write in answers: hotels and friends' places. The responses provide a better sense of the diversity of landscapes in which people find themselves day‐to‐day. To concentrate our data collection on a space more specific to our group of surveyees, we asked participants to select up to three locations on a map where they spend the most amount of their time and used the results to construct a transect connecting the most popular spaces (see Figure S3 in Supporting Information S1). This technique, referred to as local knowledge mapping, has been used in previous studies (see MacMurdo et al., 2022) and is a way to garner spatial insights in an anonymous, non‐identifiable fashion. These results indicated the common use of a path from just outside Justa Center, down past the Keys to Change Campus, and over to the entrance of the SOS (Figure 4).

#### 3.2.1 Thermal Metrics

We evaluated potential environmental exposures along this route at three pivotal times of day (approx. 7 a.m., 1 p.m., and 5 p.m.) using a biometeorological cart (MaRTy, Middel & Krayenhoff, 2019) and remotely sensed PM_2.5_ concentrations. These times were chosen for several reasons. First, they correspond with morning, lunch/midday, and afternoon commute times when local emissions are likely at their peak. Additionally, they correspond to the typical travel times to and from Justa Center (from its opening time at 7 a.m. to after dinner service) and capture the zenith sun angle to resolve maximum MRT (midday), providing a diurnal profile of potential exposures. The average air temperature (*T*_air_) along our transect throughout the day was 39.1°C (102.4°F), with a maximum of 43.3°C (109.9°F) in the early evening and a minimum of 33.7°C (92.7°F) during our morning transect. However, MRT is often deemed a more suitable measurement for human thermal exposure, as it accounts for short‐ and long‐wave radiation, representing the sum of heat exchange between an individual and their environment; MRT changes as a result of shade, the underlying surface, and other surrounding materials, allowing for hyper‐specific thermal comfort metrics (Johansson et al., 2014; Oke, 2017). Thus, compared to *T*_air_, MRT measured much higher at an average of 61.6°C (142.9°F) throughout the day along our transect, peaking at 77.1°C (170.8°F) during the midday transect. Our remaining results focus on a comparison of the measurements at our three areas of interest along the transect: Justa Center, Keys to Change Campus, and the SOS (see Figure S4 in Supporting Information S1).

**Figure 5:**
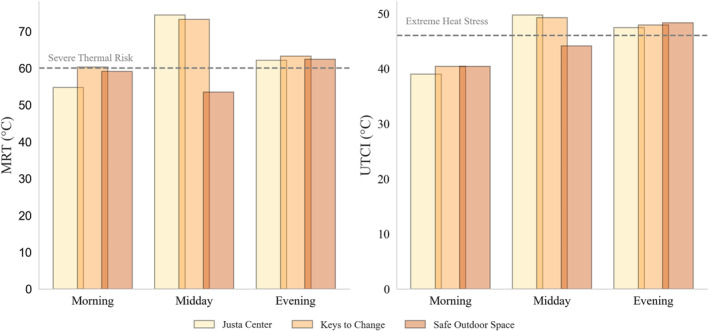
Cross‐site comparisons of mean radiant temperature (MRT) and Universal Thermal Climate Index (UTCI) across three different sites (Justa Center, Keys to Change Campus, and Safe Outdoor Space) during morning, midday, and evening time intervals. The dotted lines indicate critical thresholds for severe thermal risk (MRT ≥60°C) and extreme heat stress (UTCI ≥46°C).

Thorsson et al. (2014) established 59.4°C (138.9°F) as a MRT threshold for heat stress, a value rounded to 60°C (140°F) in subsequent studies (e.g., Chen et al., 2016; Thorsson et al., 2017). Although there may be regional differences in MRT thresholds that elicit heat stress (these studies cited focus on Shanghai), due to factors such as acclimatization and adaptation (see Baccini et al., 2008), this threshold corresponds to an extreme physiological equivalent temperature (i.e., a hot human thermal sensation level; see Lee et al., 2013). Recorded MRT is well over this 60°C (140°F) threshold during the midday and evening hours, peaking at 74.4°C (165.9°F) during the midday transect (Figure 5).

In addition to MRT, we calculated the Universal Thermal Climate Index (UTCI) for all three locations at each transect time. UTCI quantifies human thermal stress in outdoor environments by incorporating *T*_air_, wind speed, MRT, and RH into a comprehensive model that simulates heat exchange between the human body and its surroundings (Błażejczyk et al., 2013). Calculations were performed using the “pythermalcomfort” package in Python, which applies the validated UTCI algorithm (Tartarini & Schiavon, 2020). This approach accurately assesses thermal comfort or discomfort under varying climatic conditions, making it a reliable metric for evaluating heat stress in urban settings. Results indicate that, in addition to MRT, UTCI is dangerously high for both the midday and evening time frames across all three areas along our transect on a typical summer day (Figure 5). According to Błażejczyk et al. (2013), a UTCI equivalent temperature greater than 46°C (114.8°F) poses extreme heat stress risk for humans, decreasing heat loss from an individual to their environment while increasing their sweat rate. Our locations were exposed to heat above this threshold for several hours, regardless of location or underlying surface, peaking at 49.7°C (121.5°F) midday. Results indicate that evening cooling provides little respite, as MRT remains high and UTCI values are still classified as “very strong” heat stress in the early evening. Notably, the lower values at the SOS during the midday transect result from slight cloud cover, highlighting just how dependent MRT (and the subsequent UTCI calculation) is on solar radiation.

#### 3.2.2 Particulate Matter Estimates

Based on an estimation of the walking speed of the average elderly adult (see Bohannon & Williams Andrews, 2011) and an “acceptable” walk time of 15‐min found in a study by Voelkel et al. (2018), we created 0.875‐km buffers around all three areas of interest (Justa Center, Keys to Change Campus, and the SOS) in ArcGIS Pro (Version 3.3.0) to represent a reasonable walkshed for participants before extracting the PM_2.5_ values within each buffer area (Figure 4). The result is an area of approximately 3.54 square kilometers with hundreds of PM_2.5_ measurements (*n* = 345) per transect on 20 August 2024. Our estimates indicate that PM_2.5_ concentrations are highest in the morning hours before dissipating throughout the day (Table S4 in Supporting Information S1). Median values reach a maximum of 7.59 μg m^−3^ during the 7 a.m. hr while lessening to 4.19 and 3.20 μg m^−3^ during the midday and evening hours when outdoor thermal risk is most elevated.

**Figure 6:**
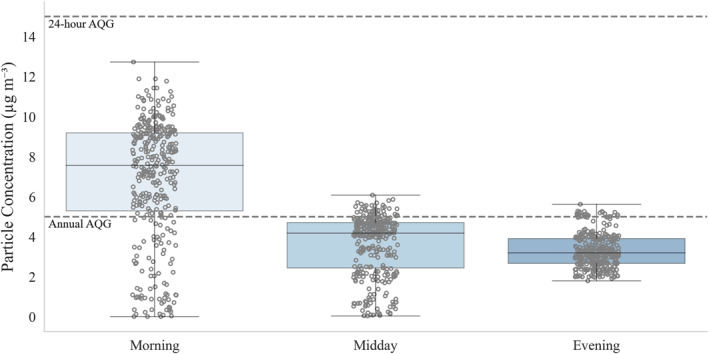
Boxplots depicting PM_2.5_ concentrations at three distinct times on 20 August 2024. Hollow circles denote each unique value (*n* = 345 per plot) within the walking buffer zone around our areas of interest. Horizontal dashed lines indicate the 24‐hr (15 μg m^−3^) and annual (5 μg m^−3^) air quality guidance suggested by the World Health Organization.

Although air quality research is diverse in recommendations for “safe” levels of PM_2.5_, the World Health Organization (WHO) sets short‐ and long‐term air quality guidance (AQG) based on a culmination of academic research: an annual PM_2.5_ level of 5 μg/m^3^ and a 24‐hr level of 15 μg/m^3^ (see World Health Organization, 2021, pp. 75–88). None of the times investigated were over the 24‐hr AQG based on our measurements (Figure 6). However, morning estimates indicate that the upper 75% of estimated PM_2.5_ concentrations surpass the annual AQG. Further, the upper end of midday and evening PM_2.5_ estimates are also above the 5 μg m^−3^ threshold. Given that this is a typical, representative day for Phoenix summer weather, these distributions indicate that our research area may pose risks for highly exposed individuals year‐round. Despite this finding, it is encouraging that acute health impacts from PM_2.5_ in the summer appear less of a concern in our study area. It is worth noting, however, that PM_2.5_ concentrations typically peak during the winter months in Phoenix as changes in human activity (e.g., indoor wood burning) and meteorological conditions create and trap more pollutants in the Valley (Brown et al., 2007). Additionally, the WHO guidelines are a culmination of scientific work, and some studies (e.g., Cakmak et al., 2018; Pinault et al., 2016; Villeneuve et al., 2015) indicate risks at lower levels than the widely‐cited AQG. Negative health outcomes at lower concentrations of PM_2.5_ are especially of concern for those with pre‐existing conditions, such as circulatory and respiratory complications, which were prevalent in this study population and indicated during surveys.

## 4 Discussion

The interactions between people experiencing homelessness and environmental hazards like heat and air pollution are a critical public health concern (Van Tol et al., 2024). Heat contributes greatly to morbidity, mortality, and hospital visits among people experiencing homelessness (Lin et al., 2024; Schwarz et al., 2022), while the impacts of air pollution among this population are less understood (DeMarco et al., 2020). Research underscores the necessity of safe and secure housing as a means to abate the risks associated with exposure to environmental hazards (Bezgrebelna et al., 2021; Gabbe et al., 2022; Hoffman et al., 2020; Shaw, 2004). Until the root challenges of homelessness are addressed (see Zhao, 2023), it remains imperative to quantify and understand these dangerous exposures for a highly exposed and vulnerable population. The thermal MRT in downtown Phoenix shows that thermal stress is unsafe for much of the day during the summer, even at the end of business hours (9 a.m.–5 p.m.) when many community resources are closed. Other studies have similar findings; many people without stable housing find themselves in spaces with elevated, unsafe temperatures (Gabbe et al., 2023; Longo et al., 2017). Air pollution can compound health risks, especially for those with pre‐existing health conditions (Sacks et al., 2011). This study estimated PM_2.5_ levels that surpass annual AQG thresholds throughout the day and approach 24‐hr AQG thresholds in the overnight to early morning hours. Previous work has similarly found elevated PM in the outdoor spaces where people experiencing homelessness travel and congregate (MacMurdo et al., 2022). While both heat and air pollution affect decision‐making, extreme heat has a greater impact on daily mobility patterns and sleeping location decisions than air pollution, underscoring the profound impact of acute thermal discomfort and the potential dangers caused by extreme heat.

### 4.1 Health: Access, Risks, and Awareness

Most survey participants demonstrated awareness of the health impacts of heat and air pollution, often a critical prerequisite to action (e.g., Bugshan et al., 2022). While heat exposure is generally easier to mitigate, few respondents felt unaffected by both heat and air pollution. We found that personalized strategies to limit exposure—such as cooling towels, hydration, shade, and rest—aligned with some of the proven best practices of low‐cost cooling strategies (Jay et al., 2021). These findings echo an earlier study in Phoenix, where Sanchez (2011) highlighted the development of personalized coping strategies among unhoused individuals.

Despite living on the street, the majority of individuals in this study had insurance and easy access to medical supplies at Justa Center; yet, some struggled to store and cool medications, such as those for asthma. Justa Center provides extra services, like a small nursing staff, that make insurance, medication, storage, and well‐being more accessible compared to the broader population. While most respondents reported not regularly using alcohol or drugs, healthcare providers at Justa Center suggested these numbers may undercount actual substance use (research suggests that over one‐third of people experiencing homelessness have substance abuse issues; Polcin, 2016). This discrepancy is significant, as alcohol and drug use can heighten health risks related to heat and air pollution exposure (Ebi et al., 2021), particularly among older individuals with preexisting conditions that necessitate medication (Moore et al., 2007). Furthermore, individuals who do not self‐identify as substance users may fail to recognize behaviors that increase their vulnerability, such as neglecting hydration or consuming alcohol during the hottest parts of the day, which can further exacerbate risks associated with environmental exposures.

### 4.2 Resource Gaps

Based on our survey, many participants believe that the city is not providing enough resources to protect them from the risks associated with heat and air pollution. People experiencing homelessness navigate a multitude of factors when determining where they stay—such as policing or harassment—which can lead to higher exposure to environmental hazards and, ultimately, negative health consequences (Gabbe et al., 2023). Although exposure to environmental hazards could be abated by access to cooled spaces, there are additional tradeoffs that people experiencing homelessness consider when choosing where they spend their time. Some of our survey participants indicated that sleeping in shelters makes them feel sick. Other studies (e.g., Zhu et al., 2023) have found that infectious diseases can spread easily within shelters—they can also be noisy, limiting sleep quality or quantity (Al‐Khalil et al., 2024; Gonzalez & Tyminski, 2020). Safety is also of concern, especially for people who are a part of already marginalized groups (e.g., Abramovich, 2017). Not to mention the highly restrictive nature of some care and shelter services that limit what and who can come into the facility (pets, partners, and belongings are common barriers to shelter access; Skinner & Rankin, 2016). After a briefing from survey proctors, most of our survey participants believe that the SOS is a positive change in Phoenix, with the caveat that initiatives meant to curb homelessness must continue to lower the barriers to entry. These realities emphasize the need for intersectional approaches to socio‐environmental issues impacting people experiencing homelessness, such as a public health lens (Bowleg, 2012).

### 4.3 Limitations and Future Work

Like any cross‐sectional study (Wang & Cheng, 2020), results represent only a small snapshot in time and are limited in both geographic and temporal scope. Although findings cannot be extrapolated to other regions, populations, and times, this study's findings set an important baseline of understanding and provide preliminary evidence justifying the need for further research that examines the intersection between people experiencing homelessness, heat, and air pollution. Biometeorological data constraints should also be considered. While we did not ask any questions directly related to wildfire impacts and concerns, these topics are of growing concern for people experiencing homelessness and is an important area of future research. The MaRTy data used to compute MRT and UTCI has an accuracy of ±10% (Middel & Krayenhoff, 2019), and our computations were limited to three times in one day, missing out on a full diurnal profile. Additionally, modeled PM_2.5_ may underestimate the most extreme concentrations given the limited training sample and the acute variability possible from natural and anthropogenic phenomena (Brown et al., 2007). Despite these limitations, our results indicate that heat and PM_2.5_ reach dangerous or unhealthy levels for much of the typical summer day in Phoenix, Arizona and pose a significant burden on older adults experiencing homelessness. Future work should include younger demographics (e.g., ages 18 to 40) and build off this study to include more cross‐sector collaborators from local policy‐making bodies to improve research design, implementation, and interpretation.

## 5 Conclusion

Most previous research has focused on urban homelessness and environmental hazards as independent issues. This study provides a cross‐sectional, mixed methods approach for identifying ways to improve the climate resiliency of older adults experiencing homelessness in Phoenix, Arizona. Results show that extreme heat in downtown Phoenix reaches levels posing significant acute concerns for human health, and long‐term outdoor exposure to moderate PM_2.5_ levels is also of concern. Further, findings reveal that both heat and air pollution influence travel and sleep location decisions (particularly heat) and contribute to health issues for many survey respondents. Most participants reported a good awareness of health risks and employed strategies to reduce exposure.

Respondents noted the high value of respite centers, like Justa Center, in providing lifesaving care and services. To support this highly vulnerable population, county‐wide service providers can similarly increase access to physical health care, education, community, and coping resources while informing clients about the exacerbated risks of extreme heat and PM_2.5_ on their underlying health vulnerabilities (and personal protective actions they can take). Additionally, expanded facility hours (i.e., staying open later), which occurred this past summer at some City of Phoenix cooling centers and Justa Center, can reduce thermal exposures during peak heat stress times. Finally, expanding transportation and item storage options can improve mobility and service access. Overall, those experiencing homelessness in our study report experiencing challenging and stressful summer conditions linked to heat and air pollution exposures and personal health risks, underscoring the value of cooled indoor spaces for reprieve, particularly those with additional social support services.

## Conflict of Interest

The authors declare no conflicts of interest relevant to this study.

## Supporting information

Supporting Information S1

## Data Availability

Survey questions and responses are publicly accessible at https://portal.edirepository.org/nis/mapbrowse?packageid=knb‐lter‐cap.723.1 (Van Tol, 2025a). Mean radiant temperature data sets can be found at https://portal.edirepository.org/nis/mapbrowse?packageid=knb‐lter‐cap.722.1 (Van Tol, 2025b). The PM_2.5_ model was created and validated using hourly outputs from the EPA (https://www.airnow.gov/), NDVI data (https://developers.google.com/earth‐engine/datasets/catalog/COPERNICUS_S2_SR_HARMONIZED), NTL data (https://developers.google.com/earth‐engine/datasets/catalog/NOAA_VIIRS_DNB_MONTHLY_V1_VCMCFG), and a host of meteorological variables (https://developers.google.com/earthengine/datasets/catalog/ECMWF_ERA5_LAND_HOURLY) from ERA5 (for more details on resolution and use, see Table S1 in Supporting Information S1). The model used to calculate PM_2.5_ in this manuscript is available on GitHub at https://github.com/zvantol/predict_pm25. Outputs of the model are publicly accessible at https://portal.edirepository.org/nis/mapbrowse?packageid=knb‐lter‐cap.721.1 (Van Tol & Peng, 2025). Universal Thermal Comfort Index (UTCI) was calculated using pythermalcomfort version 2.10.0 available for installation at https://pythermalcomfort.readthedocs.io/ (Tartarini & Schiavon, 2020). Figures were made with Matplotlib version 3.5.2 (Hunter, 2007) and seaborn version 0.11.2 (Waskom, 2021), available under their respective licenses at https://matplotlib.org/ and https://seaborn.pydata.org/. Mapping and subsequent, associated figures utilized ArcGIS Pro version 3.3.0 (Bajjali, 2023), available under the ArcGIS Pro license at https://www.esri.com/.
