# Supplementary material for: Overexposed and Understudied: Environmental Risks Among Older Adults Experiencing Homelessness in Phoenix, Arizona
Source: Geohealth. 2025 May 12;9(5):e2025GH001372. doi: 10.1029/2025GH001372 (PMC12067049; doi:10.1029/2025GH001372)
Supplement: Supplementary file 1 — Supporting Information S1 [file GH2-9-e2025GH001372-s001.docx]

*GeoHealth*

Supporting Information for

**Overexposed and Understudied: Environmental Risks Among Older Adults Experiencing Homelessness in Phoenix, Arizona**

Zachary Van Tol^1^, Ariane Middel^2^, Jennifer K. Vanos^1^, and Kristin M. Ferguson^3^

^1^School of Sustainability, Arizona State University, Tempe, Arizona, 85281

^2^School of Arts, Media and Engineering, Arizona State University, Tempe, Arizona, 85281

^3^School of Social Work, Arizona State University, Phoenix, AZ, 85006

**Introduction**

This document provides supplementary materials that support the findings and discussion presented in the manuscript. It includes additional figures and tables, which offer extended insights and visualizations of the data, as well as the full list of survey questions used in data collection. These materials are provided to enhance transparency, facilitate reproducibility, and give readers a more comprehensive understanding of the research.


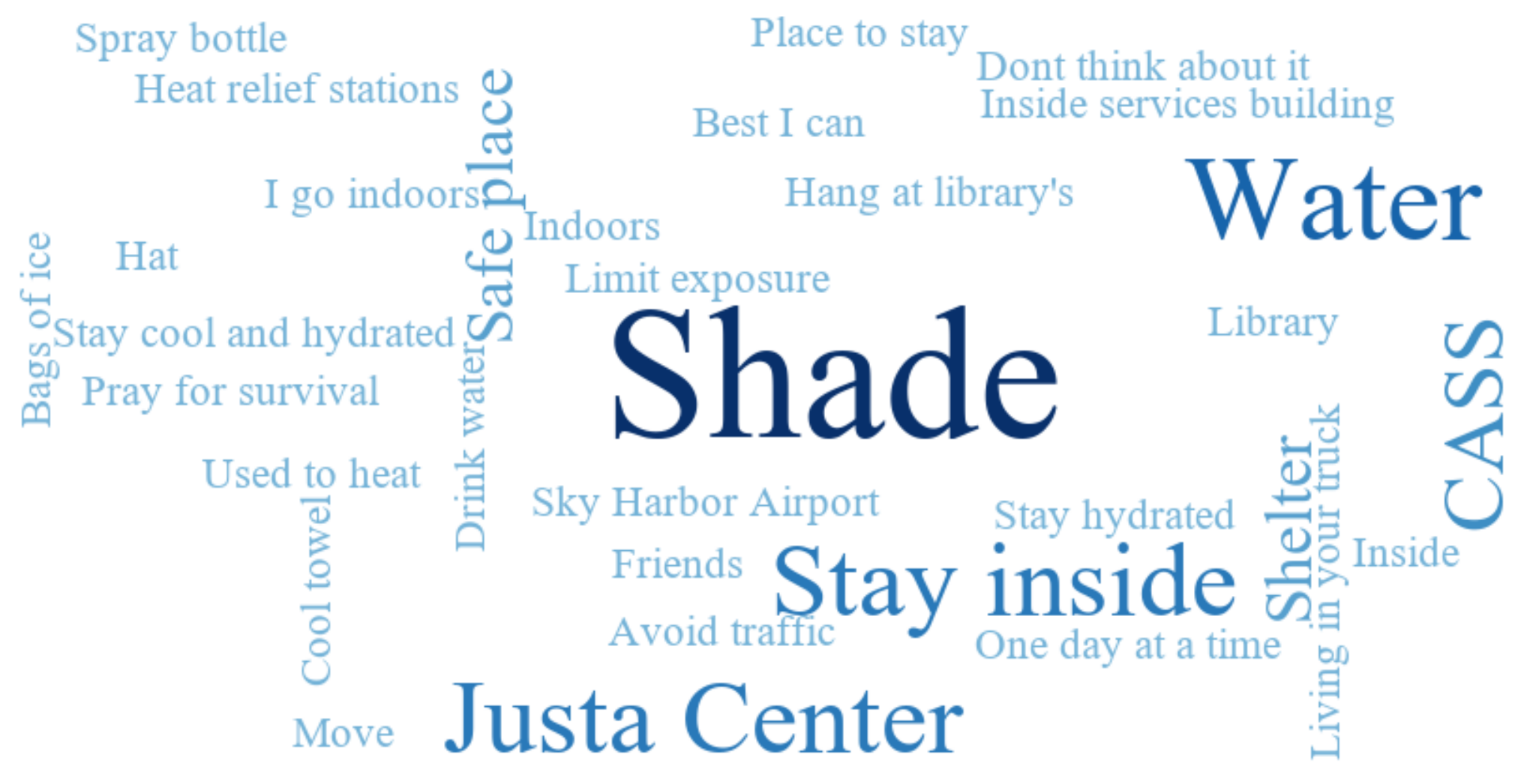


**Figure S1.** Word cloud depicts strategies for coping with heat and air pollution amongst older adults experiencing homelessness based on an open-ended survey question. Phrases occurring more frequently are represented with larger, darker text. Answers given in a list were split into multiple words/phrases to ensure proper documentation of occurrences. Some answers were changed slightly for spelling and/or to match like phrases (e.g., “Find somewhere safe” and “Finding a safe place” were both changed to “Safe place”).


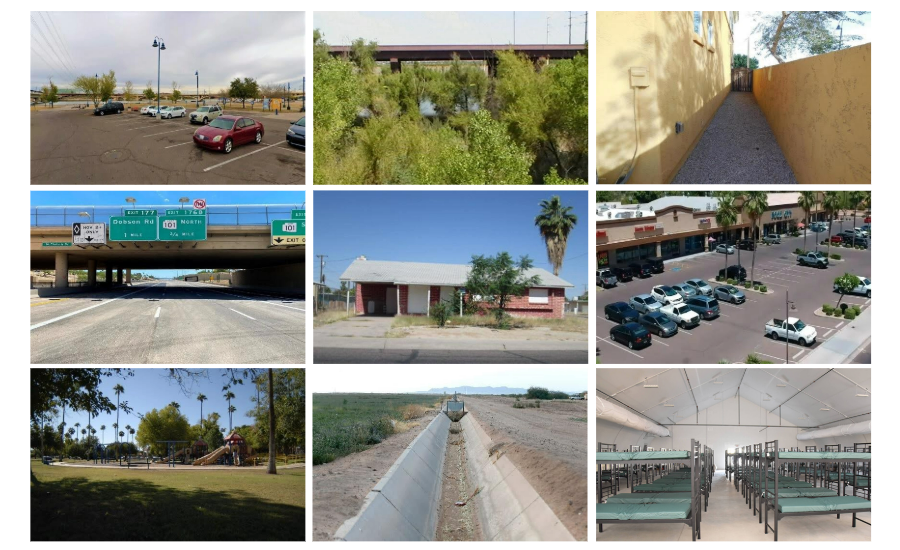


**Figure S2.** Image options as an answer to the survey question, “*In what environment do you spend most of your time?*”

**
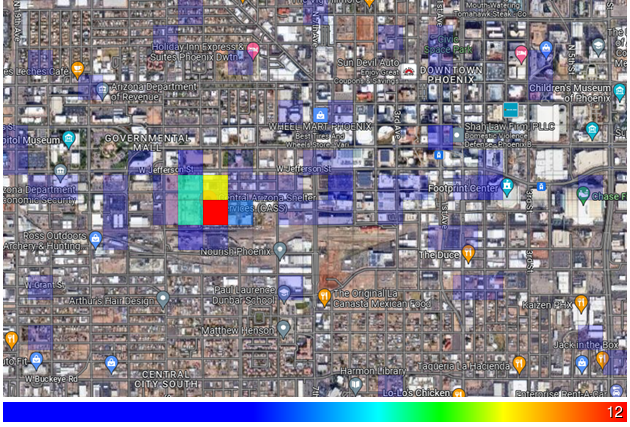
**

**Figure S3.** Respondents were presented with this map and instructed to click up to three areas they spend most of their time in. The dark red color indicates 12 clicks within that square; each square has a 25-pixel radius.


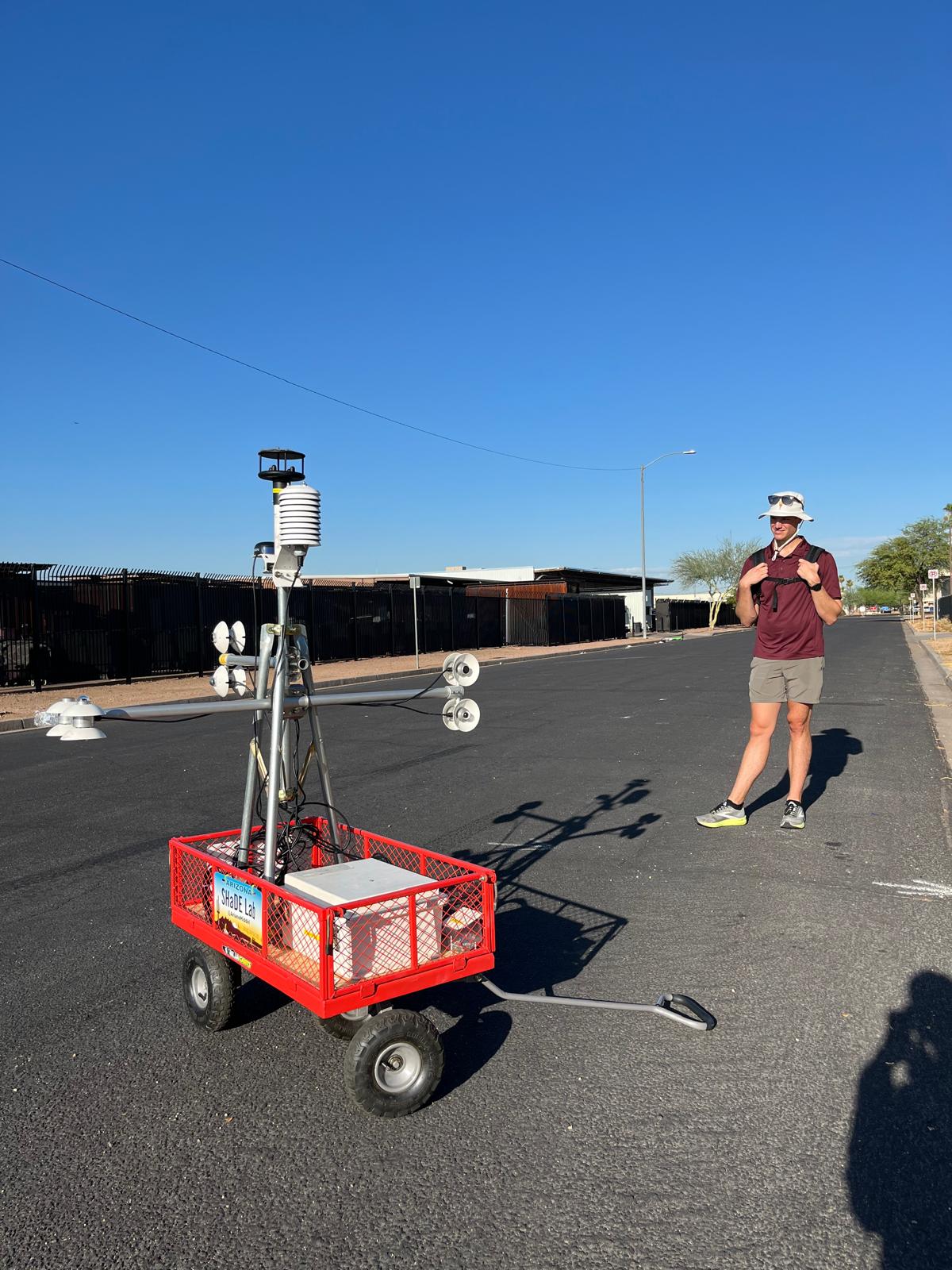

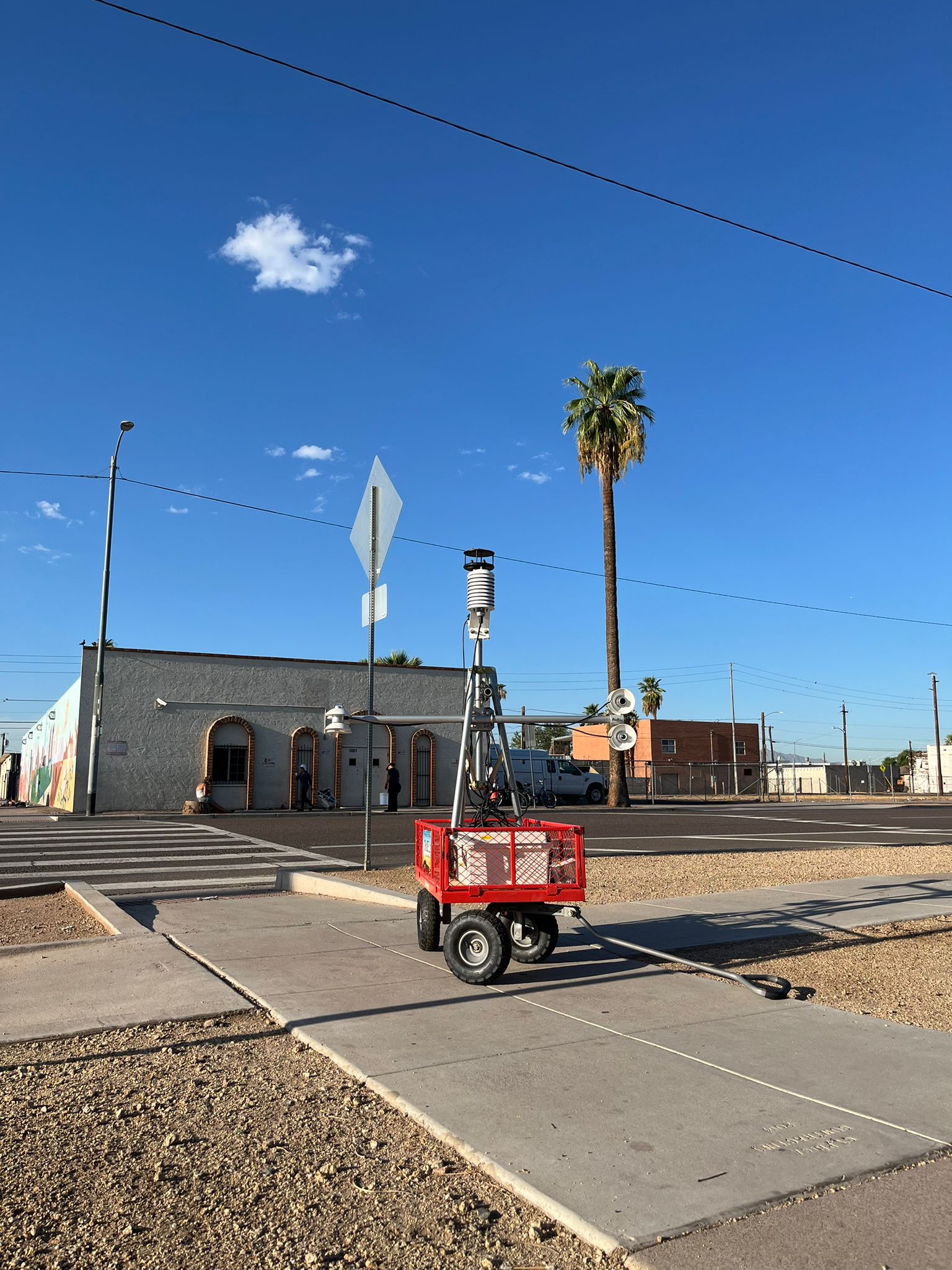

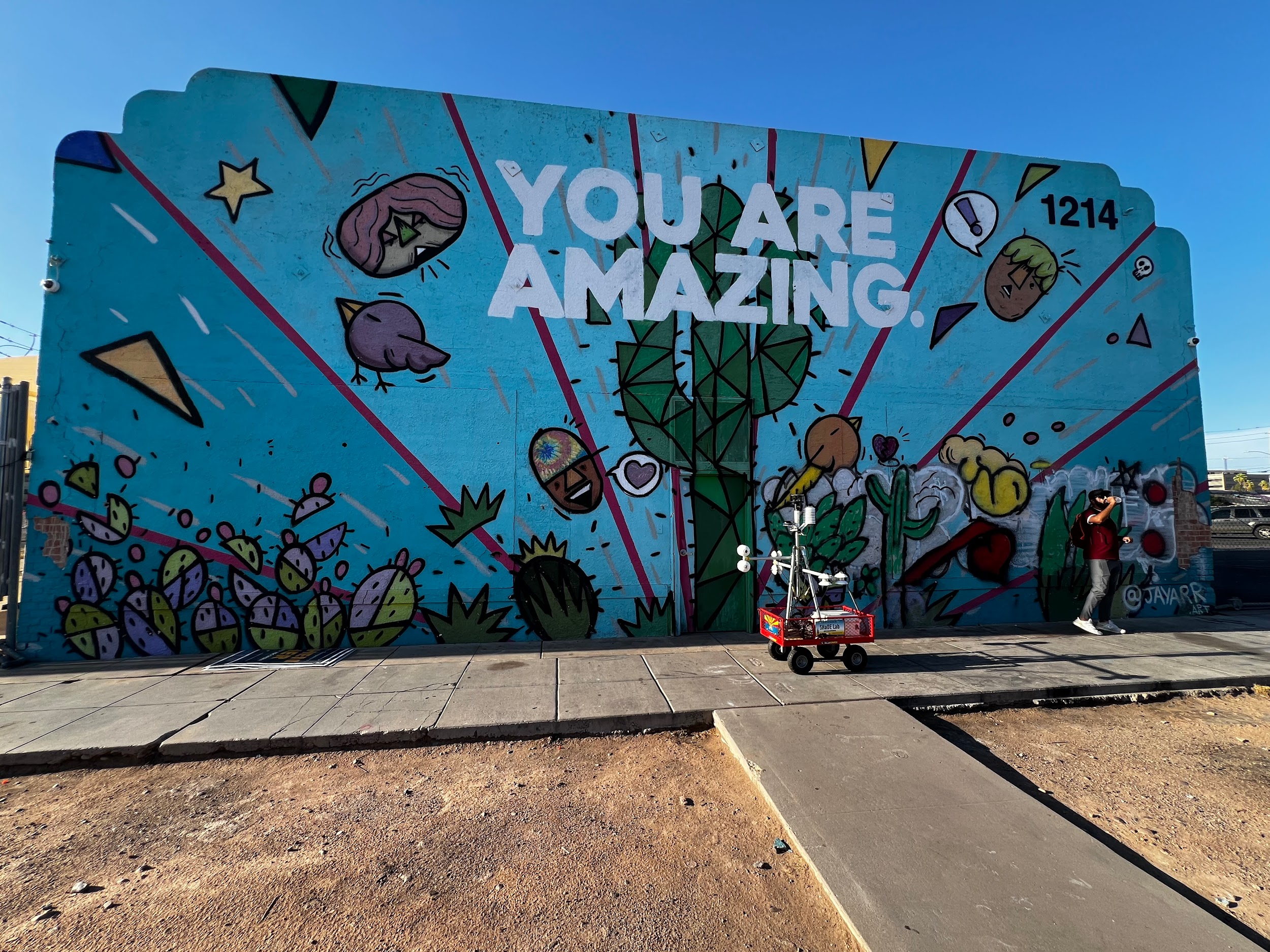


**Figure S4.** MaRTy at our three areas of interest during biometeorological transects on August 20th, 2024. From left to right: Justa Center, Keys to Change Campus, and Safe Outdoor Space.

| **Table S1.** Summary of the PM_2.5_ model inputs. | | | | |
| --- | --- | --- | --- | --- |
| *Input* | *Unit* | *Spatial Res.* | *Temporal Res.* | *Source* |
| PM_2.5_ | μg/m^3^ | — | Hourly | <https://www.airnow.gov/> |
| NDVI | — | 10 m | Monthly | <https://developers.google.com/earth-engine/datasets/catalog/COPERNICUS_S2_SR_HARMONIZED> |
| NTL | W/cm^2^/sr^-1^ | 500 m | Annually | <https://developers.google.com/earth-engine/datasets/catalog/NOAA_VIIRS_DNB_MONTHLY_V1_VCMCFG> |
| 2m T_air_ | K | 9 km | Hourly | <https://developers.google.com/earth-engine/datasets/catalog/ECMWF_ERA5_LAND_HOURLY> |
| Surface Pressure | hPa | 9 km | Hourly |  |
| Wind Speed | m/s | 9 km | Hourly |  |
| Wind Direction | degree | 9 km | Hourly |  |

| **Table S2.** Impact of transportation and belongings on accessing necessary services percentage and (count). | | | | | |
| --- | --- | --- | --- | --- | --- |
| *Survey Question* | *Never* | *Rarely* | *Sometimes* | *Often* | *Always* |
| Is transportation available to you when you want to access necessary services? | 12.5% (5) | 17.5% (7) | 27.5% (11) | 15.0% (6) | 27.5% (11) |
| Do your belongings limit your ability to access necessary services? | 45.0% (18) | 17.5% (7) | 17.5% (7) | 10.0% (4) | 10.0% (4) |

| **Table S3.** Survey respondents' opinions on resources the community provides to avoid the heat and poor air quality in percentage and (count). | | | | | |
| --- | --- | --- | --- | --- | --- |
| *Do you agree that the community provides enough resources to avoid ______?* | *Strongly disagree* | *Disagree* | *Neither agree nor disagree* | *Agree* | *Strongly agree* |
| The heat | 30.0% (12) | 12.5% (5) | 7.5% (3) | 32.5% (13) | 17.5% (7) |
| Poor air quality | 27.5% (11) | 25.0% (10) | 17.5% (7) | 22.5% (9) | 7.5% (3) |

| **Table S4.** Particle concentrations ($\mu$g m^-3^) for each transect route based on 345 observations for each transect time. | | | |
| --- | --- | --- | --- |
| *Transect Time* | *Minimum* | *Maximum* | *Median* |
| Morning (7 am) | 0.01 | 12.73 | 7.56 |
| Midday (1 pm) | 0.04 | 6.08 | 4.19 |
| Evening (5 pm) | 1.80 | 5.63 | 3.20 |

**Survey Instrument**

Below is a copy of the survey questions that were asked of the 40 respondents who participated in this research. Each participant was compensated for their time with a $25 gift card.

**Affirming Consent**

Q1: Have you read the consent form (or had it read to you) that outlines the purpose behind this study and any risks/benefits associated with it, and do you agree to this survey?

1. Yes

**Demographic and Behavioral Questions**

Q2: What year were you born?

List: ____________

Q3: What gender do you identify as?

1. Male
2. Female
3. Trans
4. Non-binary
5. Other: ____________

Q4: What race/ethnicity do you identify as?

1. Asian
2. Black/African American
3. Hispanic/Latino
4. Native American/Indigenous
5. White/Caucasian
6. Mixed race
7. Other: ____________
8. Prefer Not to Say

Q5: Are you a native Phoenix resident (i.e., born here)?

1. Yes
2. No

Q6: How long have you struggled with housing in Phoenix?

1. Less than a day
2. Days
3. Weeks
4. Months
5. Longer than a year

Q7: Have you had a psychiatric analysis or diagnosis of any mental illnesses (ex: depression, anxiety, bipolar disorder, post-traumatic stress disorder)?

1. Yes
2. No
3. Prefer not to Say

Q8: Are you able to access necessary medication if you are dealing with a mental illness (ex: Prozac)?

1. Yes
2. No
3. I don’t have a mental illness

Q9: Have you been diagnosed with any physical health conditions (ex: diabetes)?

1. Yes
2. No
3. Prefer not to Say

Q10: Are you able to access necessary medication if you are dealing with a physical health condition (ex: Insulin)?

1. Yes
2. No
3. I don’t have a physical health condition

Q11: What type of insurance do you have?

1. List: ____________
2. I’m not sure
3. I don’t have insurance

Q12: Is your insurance a barrier to accessing services for the following?

1. Mental health conditions
2. Physical health conditions
3. Both
4. Neither

Q13: Is there a physical health condition or disability preventing you from accessing services for the following?

1. Mental health conditions
2. Physical health conditions
3. Both
4. Neither

Q14: How often do you consume alcohol?

1 – Never

2 – Rarely

3 – Sometimes

4 – Almost every day

5 – Frequently

Q15: How often do you use illicit/illegal drugs?

1 – Never

2 – Rarely

3 – Sometimes

4 – Almost every day

5 – Frequently

Q16: Are you currently using alcohol or other substances to cope with being on the streets?

1. Yes
2. No
3. Prefer not to say

Q17: Do you have an addiction limiting your access to services?

1. Yes
2. No
3. I don’t have an addiction

**Movement Questions**

Q18: What is your primary (main) mode of transportation (ex: walking, biking, Uber, etc.)?

List: ____________

Q19: Is transportation available to you when you want to access necessary services?

1 – Never

2 – Rarely

3 – Sometimes

4 – Often

5 – Always

Q20: Can you safely store your belongings, or do you bring them with you?

1. I have a place to store my belongings
2. I haul my belongings with me

Q21: Do your belongings limit your ability to access necessary services?

1 – Never

2 – Rarely

3 – Sometimes

4 – Often

5 – Always

Q22: What is the primary reason you move places?

1. Climate (ex: to avoid the heat)
2. Food or water
3. Health services
4. Police or law enforcement make me move
5. Safety (for yourself, children, partner, or pets)
6. Other ____________

Q23: How much time do you spend outside, unshaded on an average day?

____________ hours

Q24: Does heat determine where you go during the day?

1 – Never

2 – Rarely

3 – Sometimes

4 – Often

5 – Always

Q25: Does air pollution determine where you go during the day?

1 – Never

2 – Rarely

3 – Sometimes

4 – Often

5 – Always

Q26: Does heat determine where you decide to sleep?

1 – Never

2 – Rarely

3 – Sometimes

4 – Often

5 – Always

Q27: Does air pollution determine where you decide to sleep?

1 – Never

2 – Rarely

3 – Sometimes

4 – Often

5 – Always

**Climate Perception Questions**

Q28: How often do you think about heat?

1 – Never

2 – Rarely

3 – Occasionally

4 – A moderate amount

5 – A great deal

Q29: How often do you think about air pollution?

1 – Never

2 – Rarely

3 – Occasionally

4 – A moderate amount

5 – A great deal

Q30: Do you feel like your environment has an impact on your health?

1 – Not at all

2 – Slightly

3 – Somewhat

4 – Moderately

5 – Extremely

Q31: How aware are you of human health risks associated with heat?

1 – Not at all

2 – Slightly

3 – Somewhat

4 – Moderately

5 – Extremely

Q32: Are you aware of the human health risks associated with air pollution?

1 – Not at all

2 – Slightly

3 – Somewhat

4 – Moderately

5 – Extremely

Q33: Has exposure to high heat ever caused you to feel sick?

1 – Never

2 – Rarely

3 – Sometimes

4 – Often

5 – Always

Q34: Has exposure to air pollution ever caused you to feel sick?

1 – Never

2 – Rarely

3 – Sometimes

4 – Often

5 – Always

Q35: Can you avoid the heat whenever you want to?

1 – Never

2 – Rarely

3 – Sometimes

4 – Often

5 – Always

Q36: Can you avoid air pollution whenever you want to?

1 – Never

2 – Rarely

3 – Sometimes

4 – Often

5 – Always

Q37: How do you survive heat and air pollution?

List: ____________

**Resource Questions**

Q38: Do you **agree** that the community provides enough resources to avoid the heat?

1 – Strongly disagree

2 – Disagree

3 – Neither agree nor disagree

4 – Agree

5 – Strongly agree

Q39: Do you **agree** that the community provides enough resources to avoid poor air quality?

1 – Strongly disagree

2 – Disagree

3 – Neither agree nor disagree

4 – Agree

5 – Strongly agree

Q40: Does the seasonal weather in Arizona impact your access to services?

1. Yes
2. No
3. I’m not sure

Q41: What environment do you typically sleep in?

1. Shelters
2. Transitional Housing
3. Safe Haven
4. Outdoors
5. On friends’ couches
6. Other ____________

Q42: Does the environment you typically sleep in make you feel sick?

1 – Never

2 – Rarely

3 – Sometimes

4 – Often

5 – Always

Q43: Are you aware of the new “safe outdoor space” (i.e., outdoor camping space, the “lot”)?

1 – Not at all

2 – Slightly

3 – Somewhat

4 – Moderately

5 – Extremely

Q44: Do you **use** the “safe outdoor space” (i.e., outdoor camping space, the “lot”)?

1. Yes
2. No
3. Prefer not to Say
4. Other ____________

Q45: Do you **want to** use the “safe outdoor space” (i.e., outdoor camping space, the “lot”)?

1. Yes
2. No
3. Prefer not to Say
4. Other ____________

Q46: The “safe outdoor space” (i.e., outdoor camping space, the “lot”) is a **positive** change.

1 – Strongly disagree

2 – Disagree

3 – Neither agree nor disagree

4 – Agree

5 – Strongly agree

Q47: The “safe outdoor space” (i.e., outdoor camping space, the “lot”) provides enough **freedom** to those who stay there.

1 – Strongly disagree

2 – Disagree

3 – Neither agree nor disagree

4 – Agree

5 – Strongly agree

Q48: The “safe outdoor space” (i.e., outdoor camping space, the “lot”) provides all the resources necessary to stay **healthy**.

1 – Strongly disagree

2 – Disagree

3 – Neither agree nor disagree

4 – Agree

5 – Strongly agree

Q49: The “safe outdoor space” (i.e., outdoor camping space, the “lot”) provides adequate **shade** and access to **air conditioning**.

1 – Strongly disagree

2 – Disagree

3 – Neither agree nor disagree

4 – Agree

5 – Strongly agree

Q50: Things the “safe outdoor space” (i.e., outdoor camping space, the “lot”) could provide to improve my quality of life.

List: ____________

**Local Knowledge Mapping**

Q51: In what environment do you spend most of your time?

1. Shelters


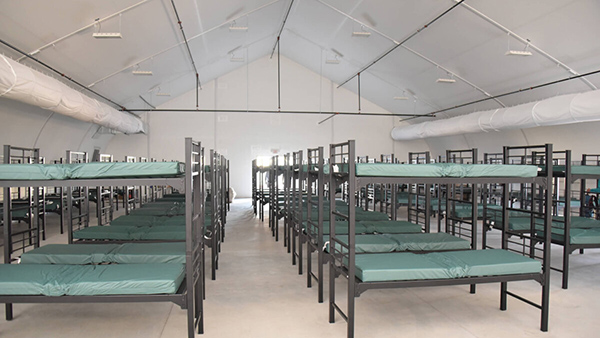


1. Abandoned Buildings


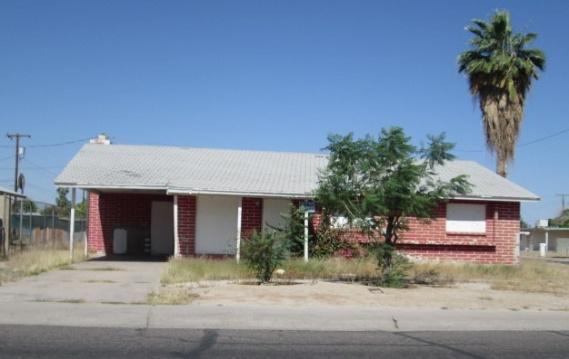


1. Alleyways


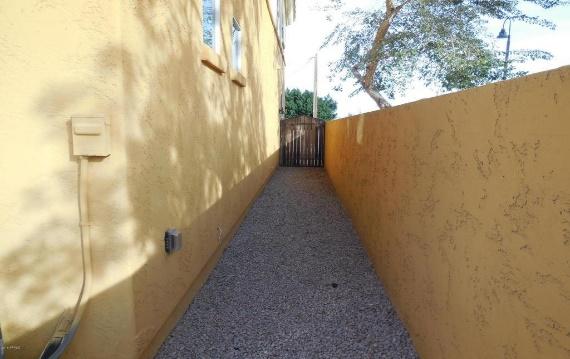


1. Canals


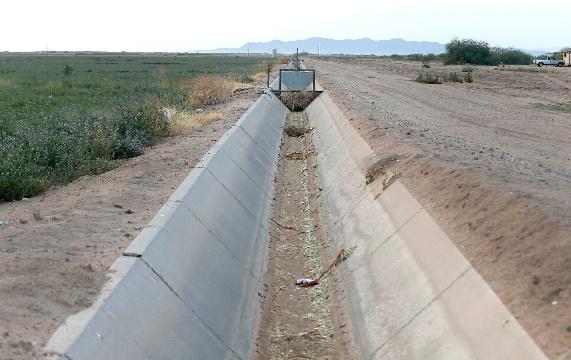


1. Residential parks


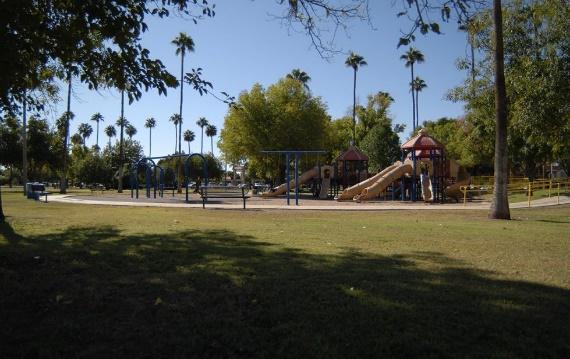


1. Under an overpass


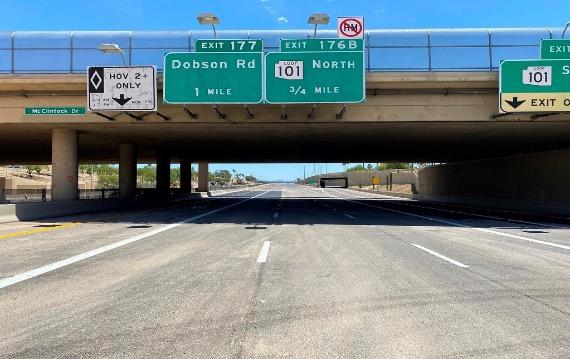


1. Parking lots


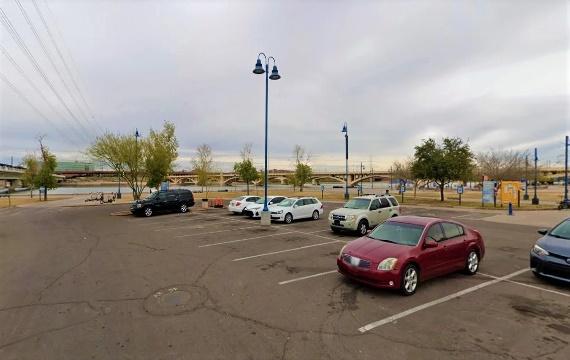


1. Shopping centers/strip malls


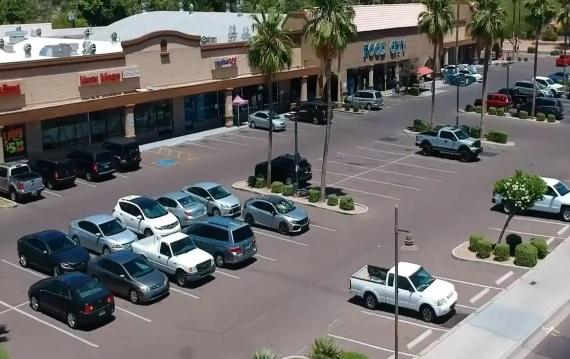


1. Vegetated areas


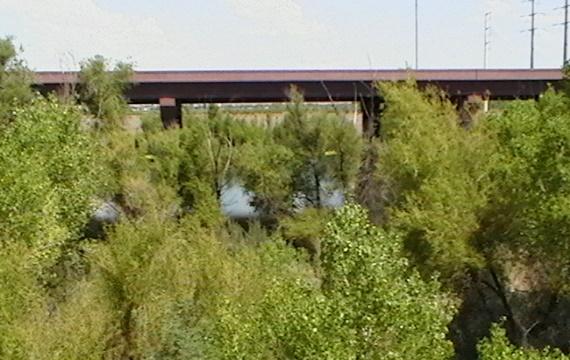


Q52: Please circle the areas (3 max) that you spend the most time in.


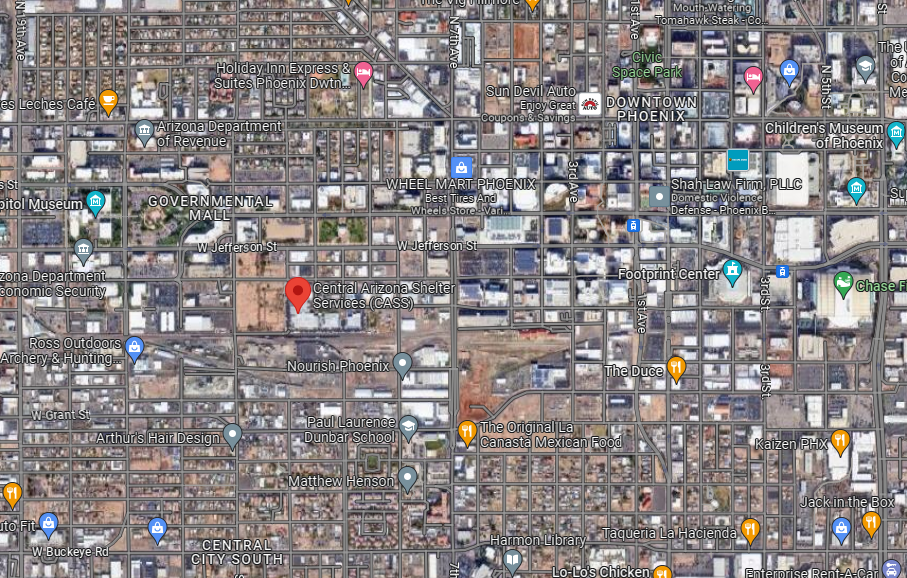


**References**

[CV 2022-010439, V000A P. McKinley ___ (SUPERIOR COURT OF ARIZONA MARICOPA COUNTY 2023). https://www.goldwaterinstitute.org/wp-content/uploads/2023/09/CV2022-010439-926-09202023.pdf](https://www.zotero.org/google-docs/?a5UXTc)

[Ebi, K. L., Capon, A., Berry, P., Broderick, C., Dear, R. de, Havenith, G., Honda, Y., Kovats, R. S., Ma, W., Malik, A., Morris, N. B., Nybo, L., Seneviratne, S. I., Vanos, J., & Jay, O. (2021). Hot weather and heat extremes: Health risks. *The Lancet*, *398*(10301), 698–708. https://doi.org/10.1016/S0140-6736(21)01208-3](https://www.zotero.org/google-docs/?a5UXTc)

[Martin, R. (2022, August 26). *The population at an Arizona homeless encampment swells but resources fall short*. NPR. https://www.npr.org/2022/08/26/1119568398/the-population-at-an-arizona-homeless-encampment-swells-but-resources-fall-short](https://www.zotero.org/google-docs/?a5UXTc)
